# Cross-Linked Self-Standing Graphene Oxide Membranes: A Pathway to Scalable Applications in Separation Technologies

**DOI:** 10.3390/membranes15010031

**Published:** 2025-01-15

**Authors:** Juan A. G. Carrio, Vssl Prasad Talluri, Swamy T. Toolahalli, Sergio G. Echeverrigaray, Antonio H. Castro Neto

**Affiliations:** 1 Centre for Advanced 2D Materials, National University of Singapore, Singapore 117546, Singapore; c2dvpt@nus.edu.sg (V.P.T.); sergio@nus.edu.sg (S.G.E.); c2dhead@nus.edu.sg (A.H.C.N.); 2 Centre for Hydrogen Innovations, National University of Singapore, E8, 1 Engineering Drive 3, Singapore 117580, Singapore; 3 Department of Materials Science and Engineering, National University of Singapore, Singapore 117575, Singapore; 4 Institute for Functional Intelligent Materials (I-FIM), National University of Singapore, Singapore 117544, Singapore

**Keywords:** graphene, metal oxide cross-linking, carbide cross-linking, membrane

## Abstract

The large-scale implementation of 2D material-based membranes is hindered by mechanical stability and mass transport control challenges. This work describes the fabrication, characterisation, and testing of self-standing graphene oxide (GO) membranes cross-linked with oxides such as Fe_2_O_3_, Al_2_O_3_, CaSO_4_, Nb_2_O_5_, and a carbide, SiC. These cross-linking agents enhance the mechanical stability of the membranes and modulate their mass transport properties. The membranes were prepared by casting aqueous suspensions of GO and SiC or oxide powders onto substrates, followed by drying and detachment to yield self-standing films. This method enabled precise control over membrane thickness and the formation of laminated microstructures with interlayer spacings ranging from 0.8 to 1.2 nm. The resulting self-standing membranes, with areas between 0.002 m^2^ and 0.090 m^2^ and thicknesses from 0.6 μm to 20 μm, exhibit excellent flexibility and retain their chemical and physical integrity during prolonged testing in direct contact with ethanol/water and methanol/water mixtures in both liquid and vapour phases, with stability demonstrated over 24 h and up to three months. Gas permeation and chemical characterisation tests evidence their suitability for gas separation applications. The interactions promoted by the oxides and carbide with the functional groups of GO confer great stability and unique mass transport properties—the Nb_2_O_5_ cross-linked membranes present distinct performance characteristics—creating the potential for scalable advancements in cross-linked 2D material membranes for separation technologies.

## 1. Introduction

Separating and purifying substances from mixtures of liquids, vapours, and gases are fundamental to several modern industrial processes. Membrane technologies have been intensively explored as energy-efficient alternatives to conventional industrial separation methods, often requiring high temperatures and costly thermal insulation systems. Among these technologies, pervaporation (PV) and vapour permeation (VP) processes have emerged as promising solutions for recovering organic solvents from aqueous solutions, offering high flux and separation factors with low capital and energy costs [1,2,3]. While PV has been widely studied for ethanol–water separation in binary mixtures, challenges like membrane fouling with direct contact [4,5] limit its application in several industries, such as beverage, for fermentation broths. Therefore, the VP process is better suited for such scenarios, reducing fouling risks by limiting direct contact. Various membrane types, including inorganic and mixed-matrix membranes, have been explored for VP applications [6,7].

At the molecular and atomic levels, membranes based on laminates of 2D materials represent a highly efficient approach to separation. These membranes integrate unique characteristics, such as nano- and angstrom-scale pore-size distributions, highly interlocked nanochannel structures, and strong physicochemical interactions with liquids and gases due to their surface charge. These properties have fundamental advantages in molecular sieving and selectivity, as evidenced by the well-established performance of graphene-based membranes in gas and liquid separations [8,9,10].

Traditional nanostructured graphene oxide (GO) membranes consist of stacked layers of graphene sheets with lateral sizes ranging from 1 to 10 μm, spaced at distances of 0.9 to 1.2 nm due to oxygenated functional groups, such as epoxide, hydroxy, and carboxy groups [9]. The predominantly random distribution of these chemical groups allows the formation of a percolative network of pristine graphene channels, which is fundamental for mass transport within the membranes [11]. However, the physicochemical and mechanical properties of laboratory-scale 2D material-based membranes often fail to meet the requirements of industrial-scale processes. To address such challenges, the use of multivalent metallic cations as cross-linking agents between GO nanosheets to improve the stability and mechanical stiffness of GO membranes has been reported in the literature [12,13,14].

Based on this concept, in this work, we explored the use of metallic oxides and carbide as cross-linking agent sources, which not only improve the mechanical stability of GO membranes but also impart additional functionalities that significantly influence their mass transport and separation performance.

Here, we introduce a new generation of self-standing graphene oxide (GO) membranes designed to meet the requirements of large-scale and industrial applications. Our fabrication methodology enables great control over membrane thickness and facilitates the formation of highly ordered laminated microstructures with interlayer distances ranging from 0.8 to 1.2 nm. By combining the selectivity of 2D laminates with a design that addresses the challenges of traditional 2D material membranes, our approach expands the potential for scalable advancements in cross-linked 2D material membranes for separation technologies.

Previous research works have reported the cross-linking effect of pure metal cations in GO [12,15]. Our work concentrates on investigating the alternative of using instead oxides and carbides. Using metallic oxides and carbides in graphene oxide composites offers several advantages over pure metals. Metal oxides and carbides are cheaper, primarily due to the lower costs associated with raw materials sources and processing. Metal oxides and carbides are also typically safer alternatives to pure metals in terms of toxicity due to their lower reactivity and limited environmental persistence. The enhanced physicochemical properties achieved by combining graphene with metal oxides are beneficial in applications like environmental remediation, photocatalysis, and electrocatalysis [16,17,18]. Their unique catalytic and electronic properties can be tailored to specific needs, and their compatibility with graphene oxide enhances the potential for synergistic effects in areas like water filtration, energy storage, and gas separation. Focusing on these materials ensures sustainability, safety, and performance without compromising functionality or economic viability.

## 2. Materials and Methods

### 2.1. Materials

Absolute Ethanol (99.8%, Fisher Scientific International Inc., Singapore, Singapore) was used to prepare ethanol/water mixtures in specific weight-to-weight (wt/wt) ratios. The density and ethanol concentration of the solutions were measured using a density metre (DMA 4500 M, Anton Paar, Graz, Austria) at 20 °C with a sample injection volume of 1.0 mL. The density metre was calibrated before sample analysis to ensure measurement accuracy.

Commercial graphene oxide (GO) in the form of aqueous paste (10% GO, Abalonyx Innovative Materials, Oslo, Norway) was used to prepare the GO suspensions and membranes. Cross-linking agents were sourced from powder forms of niobium pentoxide (Nb_2_O_5_, ≥99%, CBMM), iron (III) oxide (Fe_2_O_3_, 310,050 ≥ 99%, Sigma-Aldrich, St. Louis, MO, USA), calcium sulphate hemihydrate (CaSO_4_·0.5H_2_O, 12,090 ≥ 97%, Sigma-Aldrich), calcinated alumina (Al_2_O_3_, CT800, Almatis), and silicon carbide (SiC, Saint Gobain, Courbevoie, France). Porous plates of CaSO_4_ and CaSO_4_/Al_2_O_3_ (1:1 wt. ratio) were fabricated in-house, while SiC tubes were outsourced from Saint Gobain to be used as cross-linking agent sources and substrates for membrane casting.

### 2.2. Pervaporation and Vapour Permeation Experiments

The PV and VP experiments were conducted using custom-made cells (Figure S1, Supplementary Materials). On PV tests, the feed solutions were circulated across one side of the membrane using a peristaltic pump (Watson Marlow) at a flow rate of 15 mL min^−1^. Nitrogen gas was flowed on the opposite side of the membrane at a controlled rate of 0.1 L/min, serving as sweeping gas. The permeate was collected in a cold trap immersed in a liquid nitrogen Dewar flask (Sythware Glass Inc., Beijing, China, jacket vacuum 4 × 10^−4^ Pa). The collected permeate weight was determined using an analytical balance.

The flux (*J*), separation factor (*α*), and pervaporation separation index (PSI) were calculated by the equations:

(1) $$J=\frac{m}{A\times ∆t}$$

(2) $$\propto =\frac{y_{i}/(1-y_{i})}{x_{i}/(1-x_{i})}$$

(3) $$PSI=J\cdot \left(\alpha -1\right)$$

where *m* represents the mass transported through a membrane, *A* is the effective membrane area, ∆*t* is the time interval, and *x_i_* and *y_i_* are the mass fractions of component *i* in the feed and permeate solutions, respectively.

### 2.3. Gas Permeation Measurements

Gas permeance was measured at room temperature using the constant-volume and variable-pressure method in a self-designed experimental setup, as described in our previous work [19]. A constant pressure was applied to the feed side using a pressure controller, and the pressure increase on the permeate side was monitored using a pressure sensor until equilibrium with the feed pressure was achieved. The rate of pressure increase was recorded with a real-time pressure monitoring and recording system, and the data were used to calculate the gas permeance [19,20,21,22,23]. For gas mixture experiments, compositions were analysed using a gas chromatograph (GC-2014C, Shimadzu, Singapore).

### 2.4. Characterisation Techniques

Field emission scanning electron microscopy (FESEM, JSM-6701F, JEOL, Tokyo, Japan) equipped with energy dispersive X-ray spectroscopy (EDS) was performed to analyse membrane morphology. The membrane samples were attached to a sample holder using conductive carbon tape.

X-ray diffraction (XRD, MiniFlex 600, Rigaku, Tokyo, Japan) data were collected with CuKα radiation and Bragg–Brentano geometry. The instrument was set in step-scan mode with a step size of 0.02° and a counting time of 1 s per step.

### 2.5. Membrane Fabrication on Porous Substrates

The fabrication of cross-linked self-standing graphene oxide (GO) membranes relied on the surface interactions between metallic oxides or carbides, the cations they may release in aqueous suspension, and the functional groups of GO sheets [15]. As most metallic oxides and carbides are insoluble in water, the cross-linking interactions mainly occur directly between the particles and the GO functional groups. For instance, alumina has hydroxyl groups on its surface due to interactions with water or synthesis, which can form hydrogen bonds or covalent interactions with oxygen-containing functional groups (carboxyl, hydroxyl, and epoxy) on GO nanosheets. In the case of water-soluble metal oxides, such as CaSO_4_, metallic cations are released in aqueous solution or suspension, which subsequently promotes the formation of cross-linking sites with GO functional groups. Additionally, metallic oxides and carbides can contain a variety of impurities depending on their source, synthesis process, and post-processing treatments, such as salt, oxide, or chloride forms of alkali, alkaline earth, and transition metals. Most of these impurities are water-soluble and release cations when in aqueous solution. Thus, the specifics of the membrane formation process vary depending on the purity and type of metallic oxide or carbide used and the chosen membrane preparation procedure.

Homogeneous GO membranes were fabricated by sequentially casting multiple layers and drying each layer before the next casting step. A suspension of 0.01 mg mL^−1^ GO was prepared in water/ethanol solution (50/50 *v*/*v*) through mild sonication in a sonication bath at room temperature for 30 min. After sonication, the suspension was cast onto porous substrates of CaSO_4_, SiC, and Al_2_O_3_/CaSO_4_ (50:50 wt. ratio). The amount of suspension used per layer ranged from 0.25 to 0.30 mL cm^−2^, with a minimum of three layers per membrane. The minimal drying times for the first, second, third, and any further layers were 1, 3, 6, and 12 h, respectively, under ambient conditions. The resulting dry membranes were peeled off from the porous substrates. Membranes fabricated with three casting layers were detached by immersion in sodium dodecyl sulphate (SDS) 0.1% *w*/*v* solution in DI water, followed by washing the membranes in DI water for 1 h, as they are fragile to handling. Self-standing GO membranes were obtained with all three substrates after drying at room temperature for 24 h.

### 2.6. Membrane Fabrication with Mixed Suspensions

Mixed aqueous suspensions of GO and cross-linking agent sources were used to fabricate membranes by casting them onto polytetrafluoroethylene (PTFE) surfaces. Specifically, 0.0005 M suspensions of Nb_2_O_5_, Fe_2_O_3_ and SiC were prepared using 0.1 mg mL^−1^ GO suspension, homogenised through mild sonication for 3 h in a sonication bath. Membranes were fabricated by sequentially casting four layers onto PTFE containers, applying 0.30 mL cm^−2^ of the prepared suspension per layer. Each layer was dried at room temperature for 24 h before the next casting step. Once dried, the membranes were easily peeled off from the PTFE surface.

For comparison, self-standing GO membranes without any cross-linking agent were fabricated by casting GO suspensions onto PTFE. These reference samples are referred to as “pure GO”. The suspensions and membranes were prepared using the same concentrations and parameters described above, but the drying times for each layer were doubled.

## 3. Results and Discussions

### 3.1. Membrane Durability and Robustness

**Figure 1:**
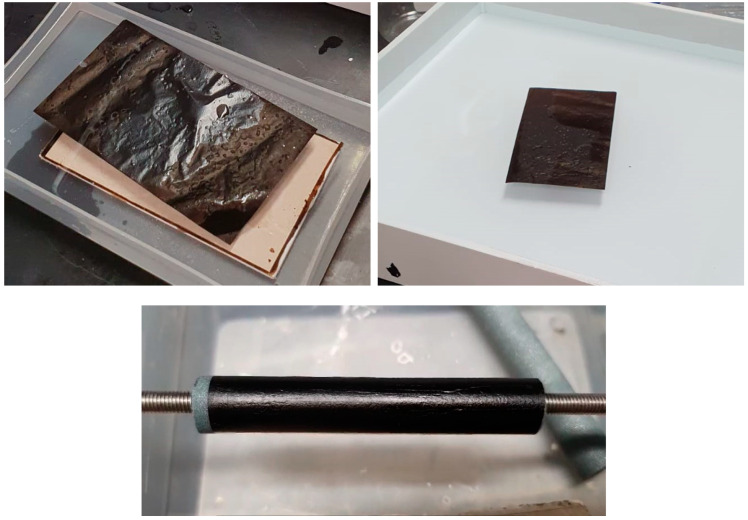
GO membrane with an area of 150 cm^2^ detaching from CaSO_4_ plate in SDS-water solution (**top-left**), membrane under washing by immersion in DI water (**top-right**), and tightly wrapped around a 10 mm diameter (**bottom**) porous ceramic substrate in a tube shape.

All membranes fabricated demonstrated exceptional chemical and mechanical robustness. They retained structural integrity even after prolonged immersion in water for several months and exhibited excellent flexibility and ease of manipulation during assembly in testing modules with different geometries. For instance, a self-standing GO membrane with an area of 150 cm^2^ was fabricated by casting onto CaSO_4_ and detached using SDS-water solution (Figure 1), followed by washing under immersion in DI water. The resulting membranes present sufficient flexibility and mechanical stability to be employed as self-standing membranes or to be tightly wrapped around porous ceramic tubes (Figure 1) or plates. The latter configuration provides additional mechanical support for separation applications operating under high pressures or flow rates. The membranes were stored in water for approximately two weeks after detachment from CaSO_4_ before assembling as self-standing membranes for PV and VP tests.

### 3.2. Macroscopic and Microscopic Overview

**Figure 2:**
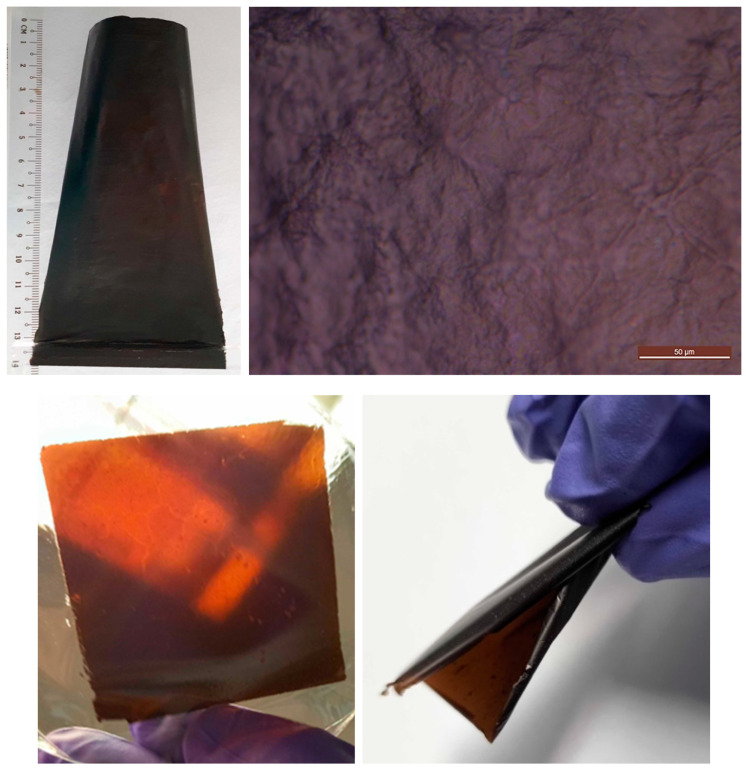
Overview of membranes with metal oxide-based cross-linking: large area self-standing membrane (**top-left**), smooth, uniform and free of cracks surface by optical microscopy (**top-right**), semi-translucent, mechanically stable, and bendable self-standing membrane (**bottom**).

A representative overview of membranes fabricated based on metal oxides/carbide as cross-linking agents and sources is presented in Figure 2. The large-area membrane cross-linked with CaSO_4_, shown in Figure 2, displays a relatively smooth, crack-free, and uniform surface, as confirmed by optical microscopy in Figure 2-top. A bendable, semi-translucent membrane (with a brown colouration) fabricated using Al_2_O_3_ is shown in Figure 2. Due to the insolubility of Al_2_O_3_ in water, some localised concentration points and defects were observed. However, the membrane remained mechanically stable and flexible, as demonstrated in Figure 2. Similar membranes were obtained with Nb_2_O_5_.

**Figure 3:**
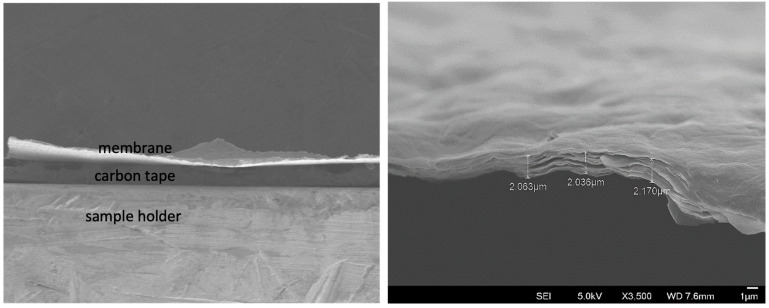
SEM image of membrane made onto SiC porous plate (**left**) and cross-sectional view (**right**) showing undulated and cracks-free membrane surface and thickness measurements.

The SEM general view in Figure 3 shows a membrane made by casting onto a SiC plate. Moreover, a cross-sectional view evidences the undulated microstructure and crack-free surface and reveals the characteristic laminated microstructure of GO membranes with an average thickness of approximately 2.09 μm.

**Figure 4:**
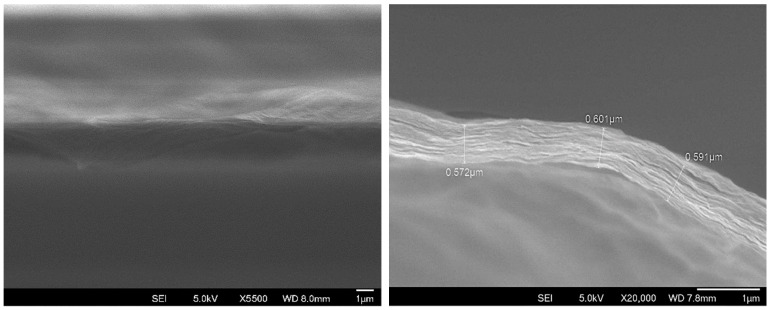
SEM image of membrane made onto CaSO_4_/Al_2_O_3_ porous plate (**left**) and cross-sectional view showing highly ordered stacking of GO layers and thickness measurements (**right**).

The membranes fabricated by casting onto CaSO_4_/Al_2_O_3_ present a smooth and undulated surface. Its laminate and crack-free continuous microstructure, featuring a highly ordered stacking of GO layers, can be observed in Figure 4, along with the thickness measurements with an average value of approximately 0.6 μm.

**Figure 5:**
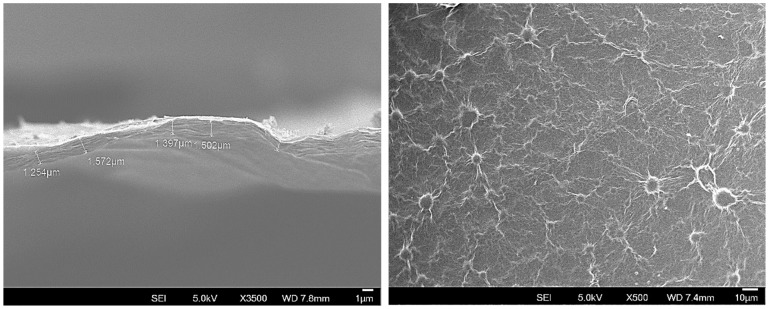
SEM cross-sectional image of membrane made with GO-Nb_2_O_5_ suspensions showing highly ordered stacking of GO layers and thickness measurements (**left**) and membrane surface with regular distribution of circular “hillock” structures and surrounding ripples (**right**).

The SEM images of a membrane prepared with GO-Nb_2_O_5_ suspensions are presented in Figure 5. The cross-sectional view presented in Figure 5-left reveals the laminated microstructure of the membrane with a thickness of approximately 1.4 μm and a well-ordered stacking of GO layers. The surface of the membrane features a regular distribution of circular “hillock” structures and surrounding ripples, with diameters ranging from about 1 μm to 10 μm, creating ripples across the surface, as detailed in Figure 5-right. Despite their size, these “hillock” structures did not lead to the formation of cracks on the membrane surface.

**Figure 6:**
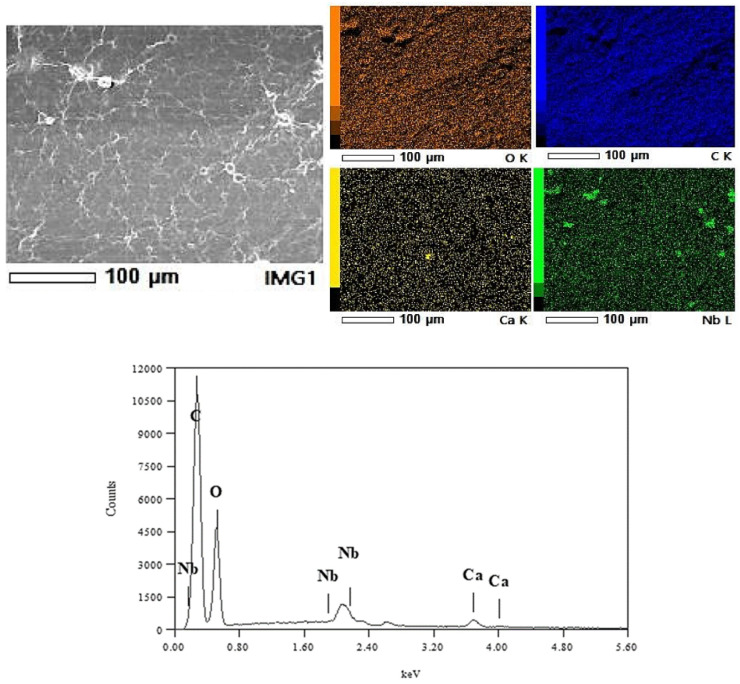
EDS analysis of the observed circular “hillock” structures on the membrane surface.

**Figure 7:**
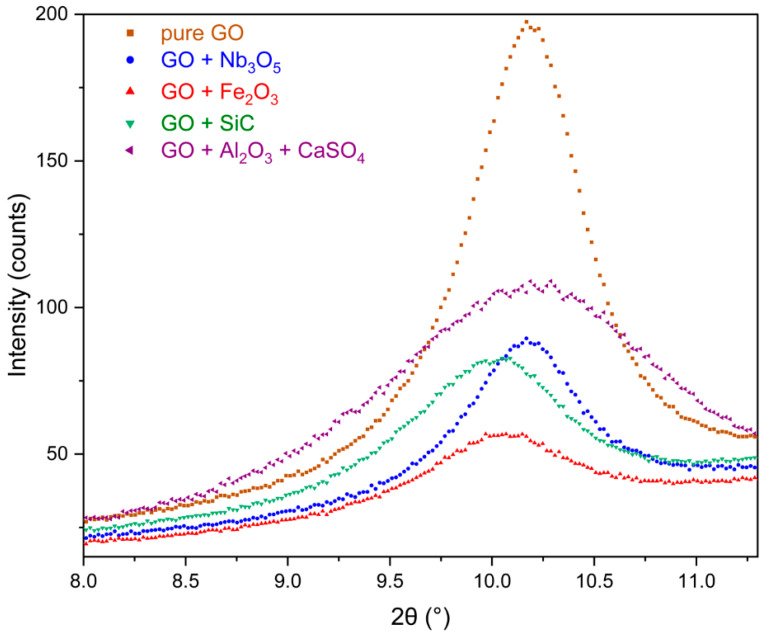
X-ray diffractograms of pure GO membranes and GO membranes with different oxides/carbide cross-linking agents or sources.

A detailed EDS analysis of the membrane surface (Figure 6) revealed niobium as the primary component of the observed circular “hillock” structures. The elements distribution analysis of a representative surface area is also shown in Figure 7, with the corresponding spectrum graph. A localised image and analysis of one of these features is presented in SI Figures S5 and S6, along with its spectrum graph.

The presence of niobium is observed across all the sample surfaces. Still, it is more concentrated in regions where elevated oxygen concentration is also observed, suggesting the presence of whole Nb_2_O_5_ particles between the GO layers, forming cross-links or physically becoming trapped during the membrane fabrication. Trace amounts of calcium were also detected, probably originating from impurities; however, its presence does not seem to have affected or compromised the smoothness or integrity of the membrane surface.

### 3.3. X-Ray Diffraction (XRD)

The X-ray diffraction patterns of pure GO membranes and those with different oxides/carbide cross-linking agents or sources are shown in Figure 7. The broad peaks around 2θ = 10.50° correspond to the (200) Bragg reflection of GO. The interplane distances for each membrane, calculated using the Bragg equation, are summarised in Table 1 (the uncertainty is ±0.04 Å). The angular positions of the diffraction peaks were determined by fitting the diffraction data with the Pearson VII function, as shown in the graphs.

### 3.4. X-Ray Photoelectron Spectroscopy (XPS)

**Figure 8:**
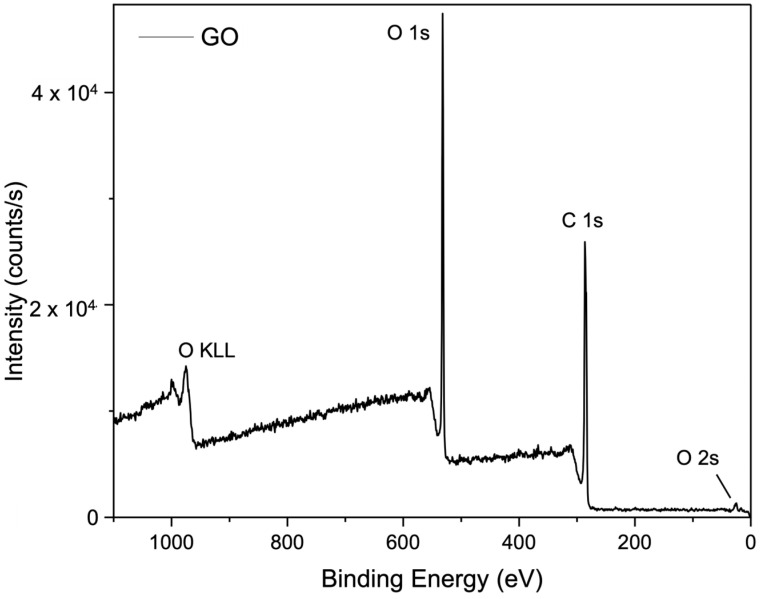
XPS survey spectrum for pure GO membrane.

X-ray photoelectron spectroscopy (XPS) was performed to investigate the detailed chemical composition of selected GO membranes, including pure GO and membranes cross-linked with CaSO_4_ + Al_2_O_3_, Nb_2_O_5_, and SiC. The survey spectrum of the pure GO membrane (Figure 8) reveals its clean composition with no impurities. The spectrum presents peaks corresponding to the binding energies of C1s, O1s, and O2s, confirming the expected elemental composition of GO.

The XPS analyses for the fabricated membranes reveal significant variations in elemental composition and binding energies, confirming the incorporation of different cross-linking agents into the membrane structure. The elemental composition variations depend on the specific cross-linking agents and their sources, highlighting how different metal oxides and carbides influence the membrane’s chemical composition, bonding environment, and structure.

**Figure 9:**
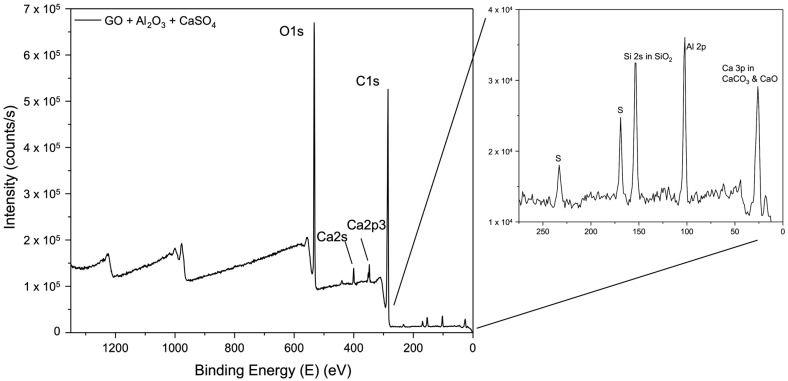
XPS survey spectrum of membrane fabricated onto CaSO_4_/Al_2_O_3_ plates. The inset presents details of the low energy range of the spectrum, revealing the presence of aluminium- and calcium-binding energies.

Figure 8 presents the XPS survey spectrum for the pure GO membranes and Figure 9 illustrates the membranes prepared on CaSO_4_/Al_2_O_3_, showing prominent peaks corresponding to O1s, Ca2s, Ca2p, and C1s binding energies. A magnified view in the range of 0 to 300 eV reveals additional peaks for Al2p and Ca3p, indicating secondary interactions involving aluminium and calcium cations, likely originated from the partial dissolution of Al_2_O_3_ and CaSO_4_ during the membrane fabrication process due to the slightly acidic nature of GO suspension. Minor impurities, such as S and SiO_2_, were also detected, probably originating from the porous ceramic substrate materials.

**Figure 10:**
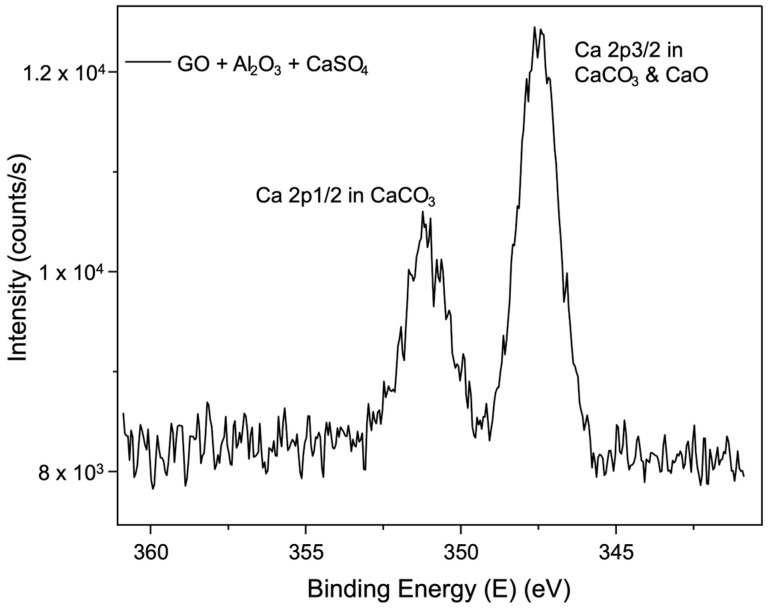
XPS spectrum of Ca2p binding energy region showing the peaks corresponding to Ca2p_3/2_ and Ca2p_1/2_.

The detailed Ca2p scan spectrum in Figure 10 further confirms the chemical states of calcium. It shows two main components due to spin–orbit coupling, with Ca2p_3_/_2_ at approximately 347.4 eV and Ca2p_1_/_2_ at around 351.1 eV. These peaks are attributed to the presence of calcium in the forms of CaCO_3_ and CaO. They display slightly higher binding energy due to the bonding with GO.

These results emphasise the role of calcium ions in promoting cross-linking between GO nanosheets and the chemical composition of the membranes. The presence of CaCO_3_ and CaO suggests that the calcium cations from CaSO_4_ contribute to forming stable cross-links, further strengthening the membrane structure.

These results emphasise the role of calcium ions in promoting cross-linking between GO nanosheets and the chemical composition of the membranes after interaction with the cross-linking agents.

**Figure 11:**
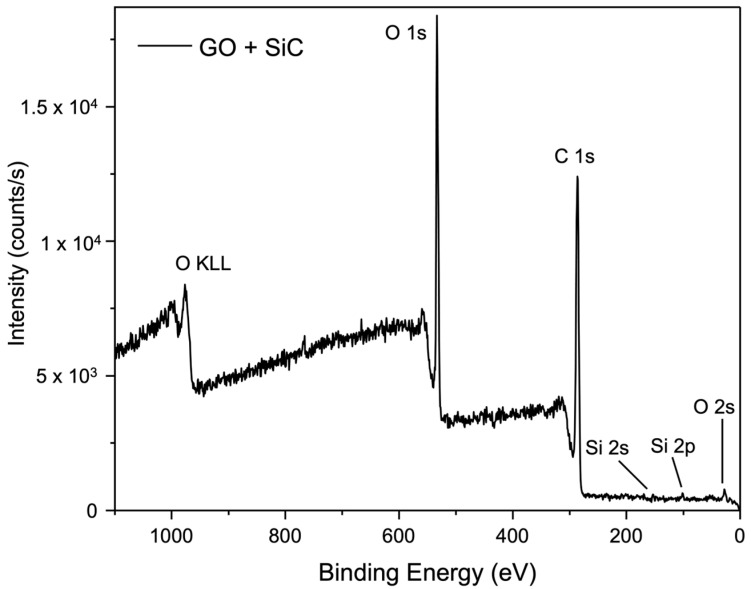
XPS survey spectrum of the membrane with SiC-based cross-linking.

**Figure 12:**
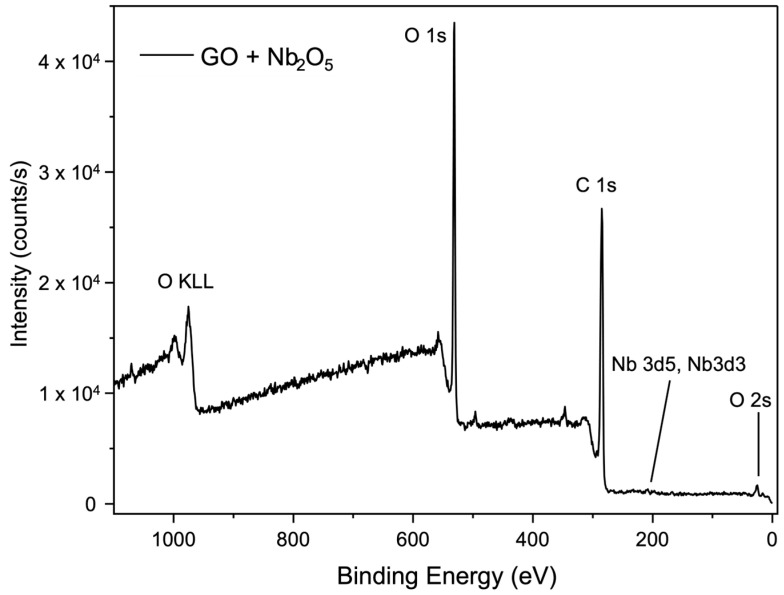
XPS survey spectrum of the membrane with Nb_2_O_5_-based cross-linking.

Figure 11 and Figure 12 present the XPS survey spectrum of membranes prepared with SiC- and Nb_2_O_5_-based cross-links. Low-intensity binding energy peaks of Si and Nb, along with the prominent peaks of C and O, were identified in both cases.

**Figure 13:**
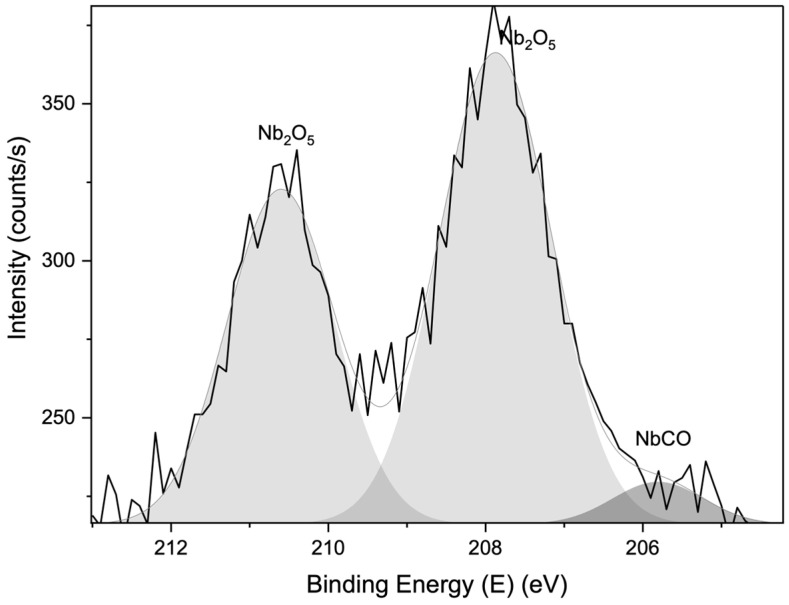
XPS spectrum of Nb3d binding energy region obtained from membrane made with Nb_2_O_5_-based cross-linking.

XPS spectra, with a large acquisition area of 50 × 100 μm, were taken from the membranes to further characterise the interaction of oxide compounds with the functional groups of GO. A qualitative comparison of the narrow C1s and O1s spectra of Nb_2_O_5_-cross-linked GO, CaSO_4_ + Al_2_O_3_-cross-linked GO, and pure GO (Figure S7) reveals differences in their peak shapes and areas, particularly in the C1s region associated with oxygen-containing functional groups [24,25,26,27,28]. In Figure 13, the XPS spectrum of the Nb3d binding energy region reveals the formation of carbide species. Considering that Nb is a transition metal, its behaviour as a cross-linking agent can be described as similar to the case of TiO_2_ nanoparticles and GO, as reported in the recent literature [29,30], where TiO_2_ reacts with the C–O and C–OH groups of GO, converting them into Ti–O–C and Ti–C species. Likewise, the formation of carbonates was observed in the case of the CaSO_4_ cross-linking-based membrane, as discussed above. The formation of carbides from the interaction of the cross-linking agents and the GO functional groups, detected by XPS peaks in the different samples, is summarised in Table 2.

Metallic oxides, such as Nb_2_O_5_, Al_2_O_3_, and CaSO_4_, can interact with graphene oxide (GO) sheets and facilitate cross-linking through various mechanisms. GO sheets are negatively charged due to oxygen-containing functional groups on their surface and edges, while metallic oxides can generate positively charged surface species in aqueous solutions [9,25,31]. These positively charged species electrostatically interact with the negatively charged GO sheets, promoting the formation of stable hybrid structures via ionic bonds or strong electrostatic attractions.

In addition, oxygen functional groups on GO can form hydrogen bonds with hydroxyl groups on the surface of metal oxide particles. These non-covalent interactions help assemble or align GO sheets in proximity. Furthermore, metallic oxides can physically anchor to defects, edges, or functional groups on GO, while metal species may also coordinate with oxygen atoms in GO to form metal-O coordination complexes [15,32,33]. This combination of mechanisms enhances interlayer binding and stabilises the structure through anchored oxide particles, offering improved mechanical strength and stability for multifunctional membranes.

A deeper XPS data analysis of cross-linked membrane samples can provide further insights into the bonding and interaction mechanisms between GO sheets and the cross-linking agents originating from the interactions of GO and metallic oxides and carbides, which will be essential to fully understanding the mechanisms, structural integrity, and performance of the membranes in separation systems.

## 4. Testing in Pervaporation and Vapour Permeation Applications

The performance of fabricated GO membranes in pervaporation and vapour permeation applications was evaluated as self-standing membranes (without any porous mechanical support). The separation processes were applied to binary solutions comprised of DI water/ethanol and DI water/methanol. For comparison, GO membranes similar to the self-standing membranes were fabricated using a well-known method for coating GO on ceramic porous substrate [5,10]. We used SiC as the porous substrate to produce these membranes, hereinafter called “coated membranes”. The membranes’ selectivity and flux were evaluated in both separation processes.

During the selectivity tests, the membranes demonstrated excellent stability and resistance to the harsh conditions of saturated vapours and liquids despite the inherently high hydrophilicity of GO. The self-standing membranes, with similar thicknesses to the coated membranes, showed a significant improvement in selectivity for the same solvent/water mixtures without requiring heating in most cases.

**Figure 14:**
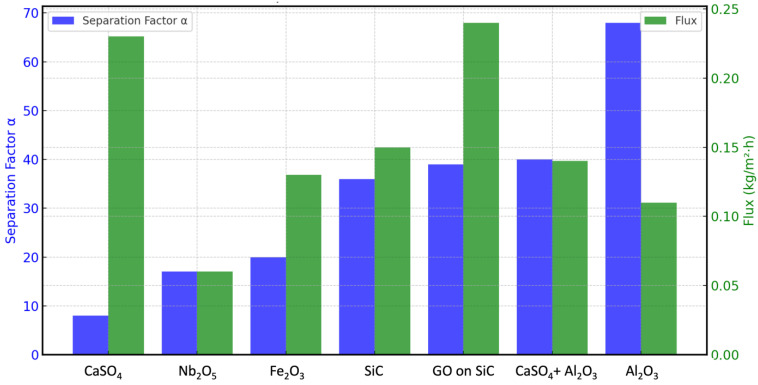
Separation factor α and flux in PV of ethanol/water mixtures with concentration 80/20 *w*/*w* at 23 °C.

**Figure 15:**
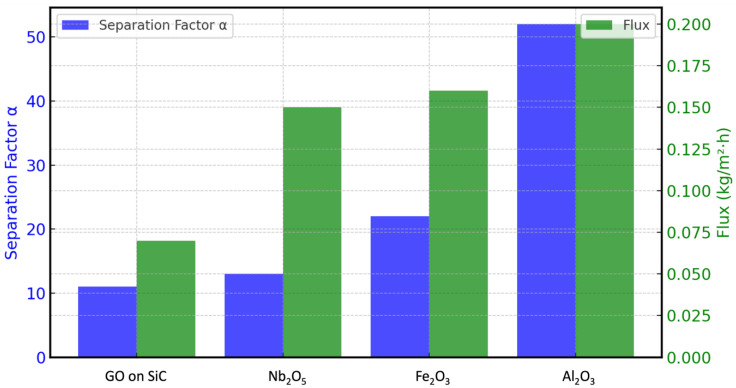
VP of ethanol/water mixtures with concentration 96/4 *w*/*w* at 40 °C.

The self-standing membranes performed well in pervaporation and vapour permeation tests for ethanol/water mixtures, as detailed in Table 3, Table 4 and Table 5 and Figure 14 and Figure 15. This improvement in selectivity emphasises the effectiveness of the self-standing membrane structures, which overcomes some of the performance limitations often encountered in coated membrane configurations. The results demonstrate the potential of these self-standing membranes for use in solvent/water separation processes, offering enhanced efficiency without the need for elevated temperatures.

For ethanol dehydration with feed concentrations exceeding 95 wt.% (the azeotrope concentration), the self-standing membranes demonstrated better performance than the coated membranes, with the selectivity and flux increased by a factor of 4.7 and 3, respectively. At lower ethanol concentrations (50 wt.%), selectivity improvements ranged from 2 to 7 times, achieving similar or higher fluxes than those detected with the coatings. Surprisingly, these performance enhancements were obtained under room temperature.

In the case of the methanol/water mixtures, the selectivity of the self-standing membranes with CaSO_4_ + Al_2_O_3_ at 40 °C was α ≈ 23, about six times higher than that of the coated membrane (α ≈ 3.8) at 50 °C, despite both membranes having similar flux values (~0.1 kg/m^2^·h).

**Figure 16:**
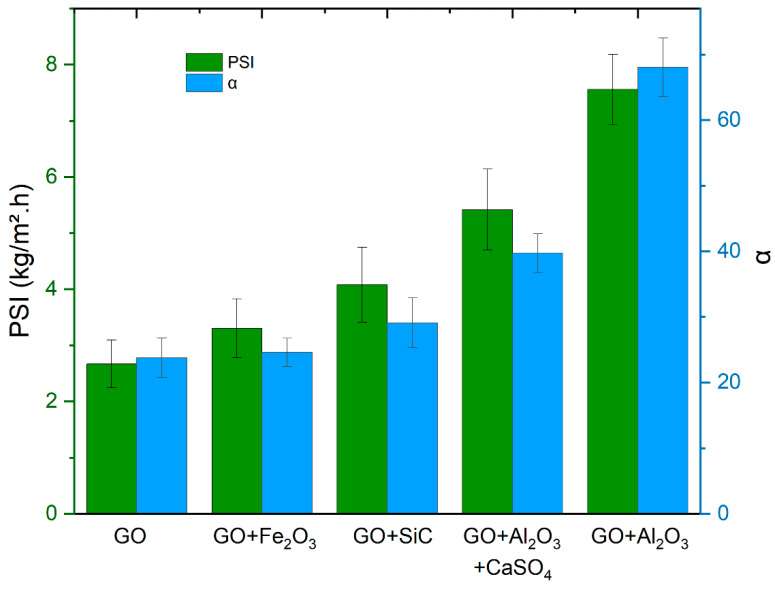
Separation factor (α) and pervaporation separation index (PSI) of membranes with and without cross-linking for PV of ethanol/water 80/20 *w*/*w* at room temperature.

Pervaporation tests for the mixture of ethanol/water 80/20 *w*/*w* at room temperature were also performed with pure GO and cross-linked self-standing membranes to investigate the effect of cross-linking on the separation performance and mechanical stability. All membranes were fabricated with the same amount of GO (20 mg) by casting four layers using the procedure described in the methodology section. The results in Table 6 show that the Al_2_O_3_ cross-linked membranes exhibited separation factors nearly three times higher, with similar or higher flux compared to the pure GO membranes. Figure 16 illustrates the superior performance of the membranes made with oxides and carbide cross-linkers compared to the pure GO membranes regarding the separation factor and pervaporation separation index. The selectivity to water over ethanol is related to the hydrophilicity of the oxides. This explains why the best performances were obtained for the membranes containing Al_2_O_3_, which is the most hydrophilic [34].

The main reason for these improvements is the ordered stacking laminate microstructure of the self-standing membranes, which addresses some structural challenges associated with coatings. Furthermore, the self-standing membranes showed increased flux and permeability in most cases, even with similar thicknesses. Based on these results, the ability to control the fabrication parameters further addresses challenges related to the scalability of GO-based membranes for various applications.

A self-standing membrane with Al_2_O_3_ + CaSO_4_ cross-linking was tested for PV of a water/methanol mixture 90/10 *w*/*w* at room temperature. The membrane showed selectivity to methanol over water with separation factor α = 2.9 ± 0.5, flux = 0.22 ± 0.1 kg/m^2^·h, and PSI = 0.36 ± 0.2 kg/m^2^·h. This result agrees with the differences in the methanol permeation behaviour to other alcohols through GO membranes described in the literature [35,36,37], which indicates that the methanol molecules can compete with the water ones due to their similar sizes and polarity.

## 5. Testing in Gas Separation Applications

Gas permeability tests were performed on self-standing cross-linked GO membranes for hydrogen (H_2_), carbon dioxide (CO_2_), and nitrogen (N_2_) gases. The membranes were subjected to high-purity H_2_, CO_2_, N_2_, and H_2_/CO_2_ gas mixture (1:1) flux using a gas permeation apparatus with a module for testing flat membranes. Porous alumina discs placed over a stainless-steel mesh were used to give mechanical support to the membranes with thicknesses ranging from 2 to 7 μm.

The membranes made with CaSO_4_+Al_2_O_3_ and with Nb_2_O_5_ cross-linking demonstrate performances beyond the upper bound, effectively limiting the trade-off between the H_2_ permeability and selectivity over CO_2_ and N_2_ [38,39]. Table 7 summarises the results of single gas permeation and ideal selectivity, with uncertainties of 16% for permeability and 7% for selectivity.

For the H_2_/CO_2_ mixture, a membrane with CaSO_4_ cross-linking showed a selectivity of 1.4 and a permeability of 21,523 Barrer, which is also a performance over the upper bound. Furthermore, all tested freestanding membranes withstood gas pressures up to at least 1.5 bar.

## 6. Conclusions

The fabricated self-standing GO membranes, with areas ranging from 0.002 m^2^ to 0.090 m^2^ and thicknesses between 0.6 μm and 20 μm, demonstrate good flexibility and chemical/physical stability. They maintained structural integrity during continuous testing in direct contact with ethanol/water and methanol/water mixtures for more than 24 h. A scalable method for the fabrication of GO-based membranes was demonstrated, and it can be extended to other 2D materials. The method was successfully applied using various metallic oxides and a carbide as cross-linking agents and sources, including Fe_2_O_3_, Al_2_O_3_, CaSO_4_, SiC, Nb_2_O_5_, a mixture of CaSO_4_/Al_2_O_3_ (50/50 *w*/*w*). The effect of these cross-linking agents in GO membranes extends far beyond enhancing mechanical stability. The interaction of the oxides with GO functional groups, particularly prominent in Nb_2_O_5_-based cross-linked membranes, suggests significant opportunities for scalable investigations into mass transport mechanisms controlled by metallic oxide cross-linking in graphene-related and other 2D materials as the membranes performed well on diverse separation applications, such as liquid, vapour, and gas separations, demonstrating their high potential.

Specifically, this work opens pathways to investigating how oxides influence the binding energies of liquid, vapours, and gas molecules in graphene-related materials. Understanding such interactions is fundamental for chemisorption and physisorption [29,40] and could promote the reversible uptake of gas molecules at near ambient conditions, improving the efficiency of separation and storage applications.

## 7. Patents

International Patent Application No. PCT/SG2024/050470: “Crosslinked Graphene-Based Membranes: Fabrication Methods, Characteristics, And Applications In Separation Processes”.

Inventors: 1. Juan Alfredo GUEVARA CARRIO (NUS), 2. VSSL Prasad TALLURI (NUS), 3. Swamy Thipperudra TOOLAHALLI (NUS), 4. Sergio GRANIERO ECHEVERRIGARAY (NUS), 5. Antonio Helio de CASTRO NETO (NUS).

Taiwan Patent Application No. 113127752: “Crosslinked Graphene-Based Membranes: Fabrication Methods, Characteristics, And Applications In Separation Processes”.

Inventors: 1. Juan Alfredo GUEVARA CARRIO (NUS), 2. VSSL Prasad TALLURI (NUS), 3. Swamy Thipperudra TOOLAHALLI (NUS), 4. Sergio GRANIERO ECHEVERRIGARAY (NUS), 5. Antonio Helio de CASTRO NETO (NUS).

## Figures and Tables

**Table 1:** Interplane distances of the pure GO membranes and GO membranes with different oxides/carbide cross-linking agents or sources.

Membrane	Angular Position 2θ (°)	d (Å)
Pure GO	10.175	8.69
GO + Nb_2_O_5_	10.170	8.69
GO + Fe_2_O_3_	10.074	8.77
GO + SiC	10.190	8.67
CaSO_4_ + Al_2_O_3_	10.348	8.54

**Table 2:** Example of XPS peak energies corresponding to carbides formed in the membranes with oxide-based cross-linking.

Cross-Link Agent (Form)	Carbides Formed	Peaks and Binding Energies
Al_2_O_3_ + CaSO_4_	Al_2_C_2_O_3_	Al2p_3/2_ at 74.95 eV C1s at 283.70eV
Al_2_O_3_ + CaSO_4_	CaCO_3_	Ca2p at 25.30 eV Ca2p_3/2_ at 347.40 eV Ca2p_1/2_ at 351.10 eV
Nb_2_O_5_ (powder in liquid)	NbC	C1s at 281.90 eV
Nb_2_O_5_ (powder in liquid)	NbCO	Nb3d_5/2_ at 205.80 eV

**Table 3:** Separation factor and flux of different membranes for PV of ethanol/water mixtures with concentration 50/50 *w*/*w* at 23 °C (uncertainties in α and flux are ±1 and ±0.06 kg/m^2^·h, respectively).

Parameter	Self-Standing Membranes with Cross-Linking		Coated Membranes
	Al_2_O_3_	CaSO_4_ + Al_2_O_3_	GO on SiC
α	33	45	16
Flux (kg/m^2^·h)	0.51	0.41	0.27

**Table 4:** Separation factor and flux of different membranes for PV of ethanol/water mixtures with concentration 80/20 *w*/*w* at 23 °C.

Parameter	Self-Standing Membranes with Cross-Linking						Coated Membranes
	Nb_2_O_5_	CaSO_4_	Fe_2_O_3_	Al_2_O_3_	SiC	CaSO_4_ + Al_2_O_3_	GO on SiC
α	17	8	20	68	36	40	39
Flux (kg/m^2^·h)	0.06	0.23	0.13	0.11	0.15	0.14	0.24

**Table 5:** Separation factor and flux of different membranes for VP of ethanol/water mixtures with concentration 96/4 *w*/*w* at 40 °C.

Parameter	Self-Standing Membranes with Cross-Linking			Coated Membranes
	Nb_2_O_5_	Fe_2_O_3_	Al_2_O_3_	GO on SiC
α	13	22	52	11
Flux (kg/m^2^·h)	0.15	0.16	0.20	0.07

**Table 6:** Separation factor, flux, and pervaporation separation index of membranes with and without cross-linking for PV of ethanol/water 80/20 *w*/*w* at room temperature.

Membrane	α	Flux (kg/m^2^·h)	PSI (kg/m^2^·h)
Pure GO	24 ± 3	0.117 ± 0.004	2.7 ± 0.4
GO + Fe_2_O_3_	25 ± 2	0.140 ± 0.005	3.3 ± 0.5
GO + SiC	29 ± 4	0.145 ± 0.003	4.1 ± 0.7
GO + Al_2_O_3_ + CaSO_4_	40 ± 3	0.140 ± 0.008	5.4 ± 0.7
GO + Al_2_O_3_	68 ± 4	0.113 ± 0.002	7.5 ± 0.6

**Table 7:** Single gas permeation and ideal selectivity for membranes with cross-linking based on Nb_2_O_5_ and Al_2_O_3_+CaSO_4_.

Membrane	H_2_ Permeability (Barrer)	CO_2_ Permeability (Barrer)	N_2_ Permeability (Barrer)	H_2_/CO_2_ Selectivity	H_2_/N_2_ Selectivity
GO + Al_2_O_3_ + CaSO_4_	90,947	32,839	33,667	2.8	2.7
GO + Nb_2_O_5_	23,398	6443	7779	3.6	3.0

## Data Availability

The datasets generated and/or analysed during the current study are available from the corresponding author upon reasonable request.

## Supplementary Materials

The following supporting information can be downloaded at: https://www.mdpi.com/article/10.3390/membranes15010031/s1, Figure S1: Vapour permeation (VP) stainless steel cell, pervaporation acrylic cell and experimental setup for simultaneous multiple cell analysis; Figure S2: Self-standing GO membranes fabricated onto CaSO_4_ wrapped around a tube with 10 mm diameter and around a tube with 25 mm diameter inside the pervaporation module; Figure S3: SEM image of membrane made onto SiC porous plate; Figure S4: SEM image of a cross-section of a membrane made onto CaSO_4_/Al_2_O_3_ porous plate; Figure S5: SEM image of a membrane made with GO-Nb_2_O_5_; Figure S6: EDS analysis of membrane sample with Nb_2_O_5_ showing the main components of near-circular objects regularly distributed all over the membrane; Figure S7: Qualitative comparison of the C1s and O1s spectra for samples with Nb_2_O_5_-based and CaSO_4_ + Al_2_O_3_-based cross-linking, as well as pure GO.

## References

[1] O’brien D.J., Roth L.H., McAloon A.J. (2000). Ethanol production by continuous fermentation–pervaporation: A preliminary economic analysis. J. Membr. Sci..

[2] Liu G., Wei W., Jin W. (2014). Pervaporation Membranes for Biobutanol Production. ACS Sustain. Chem. Eng..

[3] Si Z., Shan H., Hu S., Cai D., Qin P. (2018). Recovery of ethanol via vapor phase by polydimethylsiloxane membrane with excellent performance. Chem. Eng. Res. Des..

[4] Xue C., Liu F., Xu M., Zhao J., Chen L., Ren J., Bai F., Yang S. (2016). A novel in situ gas stripping-pervaporation process integrated with acetone-butanol-ethanol fermentation for hyper n-butanol production: Butanol production by an efficient recovery strategy. Biotechnol. Bioeng..

[5] Carrio J.A.G., Talluri V.P., Toolahalli S.T., Echeverrigaray S.G., Neto A.H.C. (2023). Gas stripping assisted vapour permeation using graphene membrane on silicon carbide for ethanol recovery. Sci. Rep..

[6] Hu S., Guan Y., Cai D., Li S., Qin P., Karim M.N., Tan T. (2015). A novel method for furfural recovery via gas stripping assisted vapor permeation by a polydimethylsiloxane membrane. Sci. Rep..

[7] Yakovlev A.V., Shalygin M.G., Matson S.M., Khotimskiy V.S., Teplyakov V.V. (2013). Separation of diluted butanol–water solutions via vapor phase by organophilic membranes based on high permeable polyacetylenes. J. Membr. Sci..

[8] Nair R.R., Wu H.A., Jayaram P.N., Grigorieva I.V., Geim A.K. (2012). Unimpeded Permeation of Water Through Helium-Leak–Tight Graphene-Based Membranes. Science.

[9] Hong S., Constans C., Martins M.V.S., Seow Y.C., Carrió J.A.G., Garaj S. (2017). Scalable Graphene-Based Membranes for Ionic Sieving with Ultrahigh Charge Selectivity. Nano Lett..

[10] Shin Y., Taufique M.F.N., Devanathan R., Cutsforth E.C., Lee J., Liu W., Fifield L.S., Gotthold D.W. (2019). Highly Selective Supported Graphene Oxide Membranes for Water-Ethanol Separation. Sci. Rep..

[11] Talyzin A.V., Hausmaninger T., You S., Szabó T. (2014). The structure of graphene oxide membranes in liquid water, ethanol and water–ethanol mixtures. Nanoscale.

[12] Park S., Lee K.-S., Bozoklu G., Cai W., Nguyen S.T., Ruoff R.S. (2008). Graphene Oxide Papers Modified by Divalent Ions—Enhancing Mechanical Properties via Chemical Cross-Linking. ACS Nano.

[13] Liu T., Yang B., Graham N., Yu W., Sun K. (2017). Trivalent metal cation cross-linked graphene oxide membranes for NOM removal in water treatment. J. Membr. Sci..

[14] Huang L., Zhang M., Li C., Shi G. (2015). Graphene-Based Membranes for Molecular Separation. J. Phys. Chem. Lett..

[15] Yeh C.-N., Raidongia K., Shao J., Yang Q.-H., Huang J. (2014). On the origin of the stability of graphene oxide membranes in water. Nat. Chem..

[16] Moharana S., Satpathy S.K., Nguyen T.A., Maharana T. (2025). Graphene–Metal Oxide Composites: Synthesis, Properties, and Applications.

[17] Akram M.Y., Ashraf T., Jagirani M.S., Nazir A., Saqib M., Imran M. (2024). Recent Advances in Graphene-Based Single-Atom Photocatalysts for CO_2_ Reduction and H2 Production. Catalysts.

[18] Kumar S.P., Sharafudeen P.C., Elumalai P. (2023). High entropy metal oxide@graphene oxide composite as electrocatalyst for green hydrogen generation using anion exchange membrane seawater electrolyzer. Int. J. Hydrogen Energy.

[19] Carrio J.A., Echeverrigaray S.G., Talluri V., Sudhakaran D.P., Gan H.T., Gardenö D., Friess K., Neto A.H.C. (2024). Performance of GO laminated membranes in H_2_/CO_2_ separation as a function of the membrane thickness. Int. J. Hydrogen Energy.

[20] Kim H.W., Yoon H.W., Yoon S.-M., Yoo B.M., Ahn B.K., Cho Y.H., Shin H.J., Yang H., Paik U., Kwon S. (2013). Selective Gas Transport Through Few-Layered Graphene and Graphene Oxide Membranes. Science.

[21] Uchytil P., Schauer J., Petrychkovych R., Setnickova K., Suen S.Y. (2011). Ionic liquid membranes for carbon dioxide–methane separation. J. Membr. Sci..

[22] Friess K., Lanč M., Pilnáček K., Fíla V., Vopička O., Sedláková Z., Cowan M.G., McDanel W.M., Noble R.D., Gin D.L. (2017). CO_2_/CH_4_ separation performance of ionic-liquid-based epoxy-amine ion gel membranes under mixed feed conditions relevant to biogas processing. J. Membr. Sci..

[23] Bouša D., Friess K., Pilnáček K., Vopička O., Lanč M., Fónod K., Pumera M., Sedmidubský D., Luxa J., Sofer Z. (2017). Thin, High-Flux, Self-Standing, Graphene Oxide Membranes for Efficient Hydrogen Separation from Gas Mixtures. Chem.—Eur. J..

[24] Al-Marri A.H., Janene F., Moulahi A., Mogharbel A.T., Al-Farraj E.S., Al-Mohaimeed A.M., Mjejri I. (2023). Enhanced photocatalytic properties of the Nb_2_O_5_/rGO for the degradation of methylene blue. Ionics.

[25] Soomro F., Memon F.H., Khan M.A., Iqbal M., Ibrar A., Memon A.A., Lim J.H., Choi K.H., Thebo K.H. (2023). Ultrathin Graphene Oxide-Based Nanocomposite Membranes for Water Purification. Membranes.

[26] Zhang W., Xu H., Xie F., Ma X., Niu B., Chen M., Zhang H., Zhang Y., Long D. (2022). General synthesis of ultrafine metal oxide/reduced graphene oxide nanocomposites for ultrahigh-flux nanofiltration membrane. Nat. Commun..

[27] Chen X., Wang X., Fang D. (2020). A review on C1s XPS-spectra for some kinds of carbon materials. Fuller. Nanotub. Carbon Nanostruct..

[28] Kovtun A., Jones D., Dell’Elce S., Treossi E., Liscio A., Palermo V. (2019). Accurate chemical analysis of oxygenated graphene-based materials using X-ray photoelectron spectroscopy. Carbon.

[29] Shiraz H.G., Tavakoli O. (2017). Investigation of graphene-based systems for hydrogen storage. Renew. Sustain. Energy Rev..

[30] Bajestani Z.G., Yürüm A., Yürüm Y. (2016). Significant improvement in the hydrogen storage capacity of a reduced graphene oxide/TiO_2_ nanocomposite by chemical bonding of Ti–O–C. RSC Adv..

[31] Hong W.G., Kim B.H., Lee S.M., Yu H.Y., Yun Y.J., Jun Y., Lee J.B., Kim H.J. (2012). Agent-free synthesis of graphene oxide/transition metal oxide composites and its application for hydrogen storage. Int. J. Hydrogen Energy.

[32] Pandey R.P., Kallem P., Hegab H.M., Rasheed P.A., Banat F., Hasan S.W. (2022). Cross-linked laminar graphene oxide membranes for wastewater treatment and desalination: A review. J. Environ. Manag..

[33] Tiwary S.K., Singh M., Chavan S.V., Karim A. (2024). Graphene oxide-based membranes for water desalination and purification. Npj 2D Mater. Appl..

[34] Li Z., Huang Z., Xie N., Gao X., Fang Y., Zhang Z. (2018). Preparation of Al_2_O_3_-coated expanded graphite with enhanced hydrophilicity and oxidation resistance. Ceram. Int..

[35] Liu R., Arabale G., Kim J., Sun K., Lee Y., Ryu C., Lee C. (2014). Graphene oxide membrane for liquid phase organic molecular separation. Carbon.

[36] Iakunkov A., Talyzin A.V. (2020). Swelling properties of graphite oxides and graphene oxide multilayered materials. Nanoscale.

[37] Dahanayaka M., Chew J.W. (2022). Organic Solvent Permeation through Negatively Charged Graphene Oxide Membranes. ACS Sustain. Chem. Eng..

[38] Robeson L.M. (1991). Correlation of separation factor versus permeability for polymeric membranes. J. Membr. Sci..

[39] Robeson L.M. (2008). The upper bound revisited. J. Membr. Sci..

[40] Ao Z., Dou S., Xu Z., Jiang Q., Wang G. (2014). Hydrogen storage in porous graphene with Al decoration. Int. J. Hydrogen Energy.

