# Supplementary material for: Cross-Linked Self-Standing Graphene Oxide Membranes: A Pathway to Scalable Applications in Separation Technologies
Source: Membranes (Basel). 2025 Jan 15;15(1):31. doi: 10.3390/membranes15010031 (PMC11766786; doi:10.3390/membranes15010031)
Supplement: Supplementary file 1 [file membranes-15-00031-s001.zip › membranes-3405534-supplementary.pdf]

Supplementary Materials: Cross-Linked Self-Standing Graphene Oxide Membranes: A Pathway to Scalable Applications in Separation Technologies

Juan A. G. Carrio [1,2,*], Vssl Prasad Talluri [1], Swamy T. Toolahalli [1], Sergio G. Echeverrigaray [1,2] and Antonio H. Castro Neto [1,2,3,4]

1 Centre for Advanced 2D Materials, National University of Singapore, Singapore 117546, Singapore; c2dvpt@nus.edu.sg (V.P.T.); sergio@nus.edu.sg (S.G.E.); c2dhead@nus.edu.sg (A.H.C.N.)

2 Centre for Hydrogen Innovations, National University of Singapore, E8, 1 Engineering Drive 3,
Singapore 117580, Singapore

3 Department of Materials Science and Engineering, National University of Singapore, Singapore 117575, Singapore

4 Institute for Functional Intelligent Materials (I-FIM), National University of Singapore, Singapore 117544, Singapore

* Correspondence: juan.carrio@nus.edu.sg

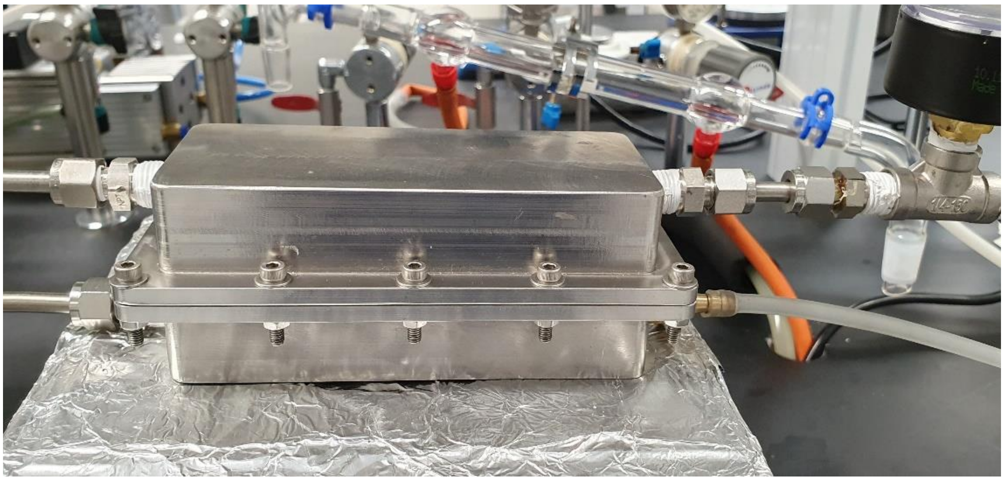

(**a**)

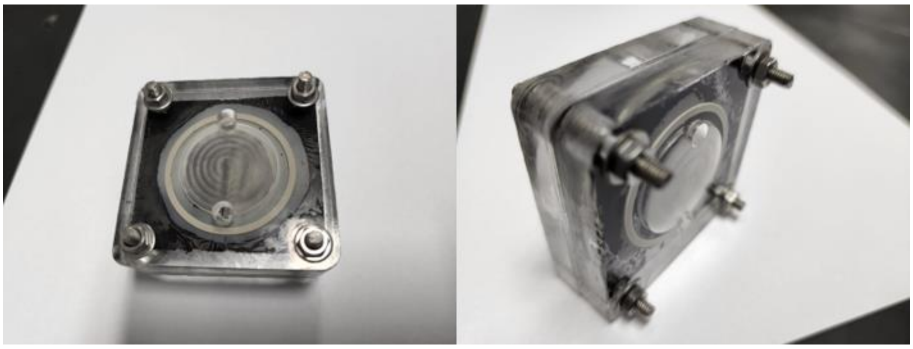

(**b**)

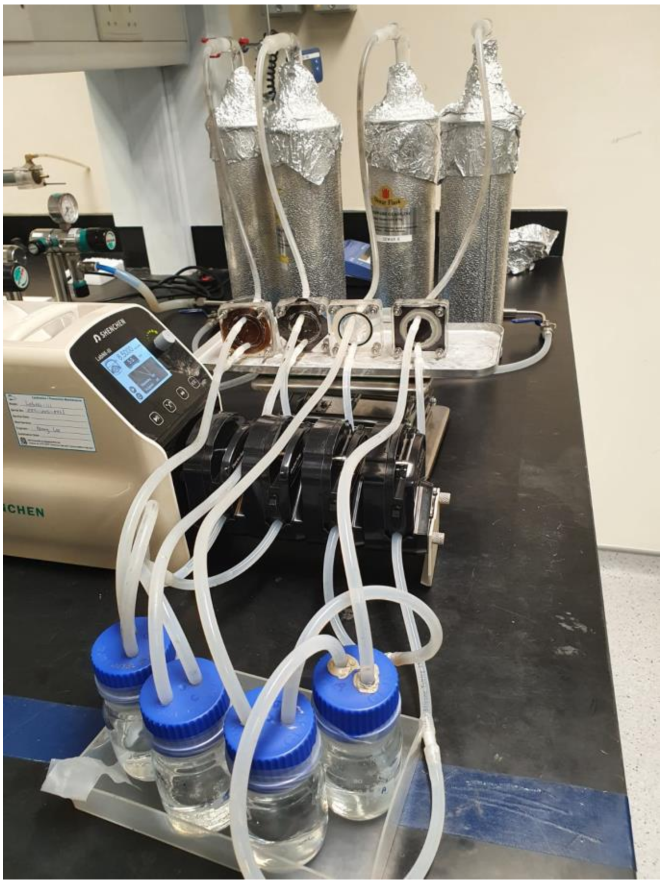

(**c**)

Figure S1. (**a**) Vapour permeation (VP) stainless steel cell, (**b**) pervaporation acrylic cell, and (**c**) experimental setup for simultaneous multiple cell analysis.

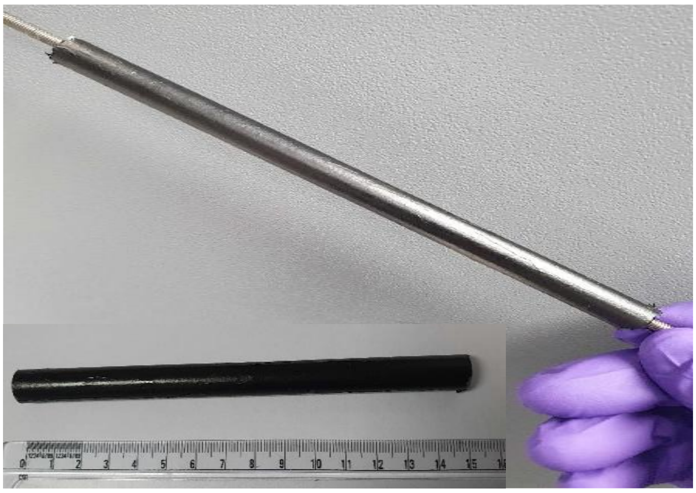

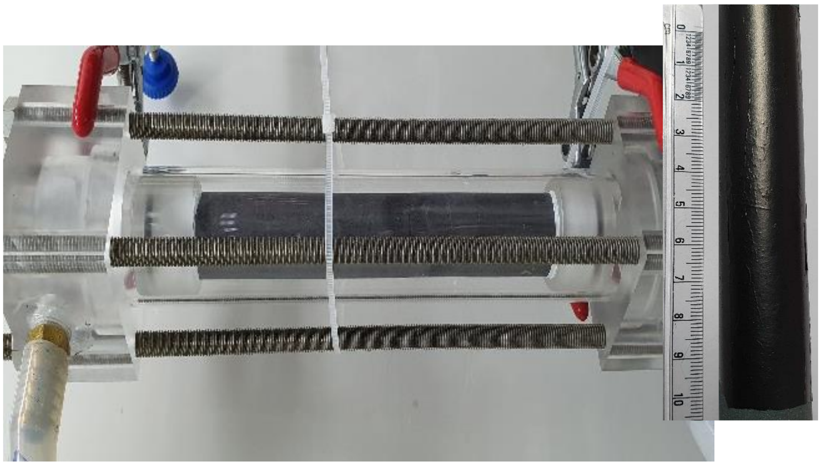

Figure S2. Self-standing GO membranes fabricated onto $CaSO_4$ wrapped around a tube with 10 mm diameter (**top**) and around a tube with 25 mm diameter ready to be tested inside the pervaporation module (**bottom**).

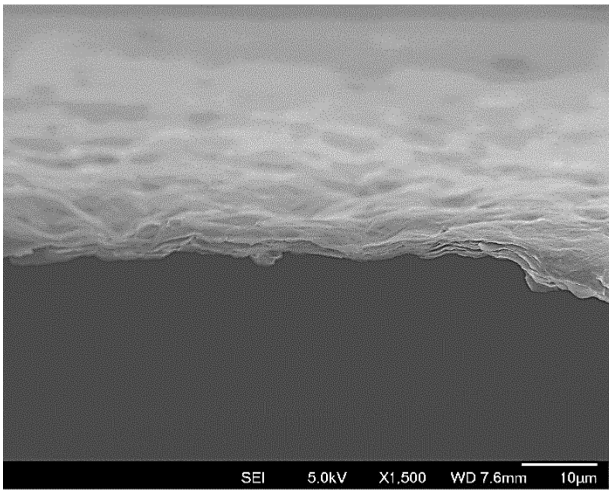

Figure S3. SEM image of membrane made onto SiC porous plate.

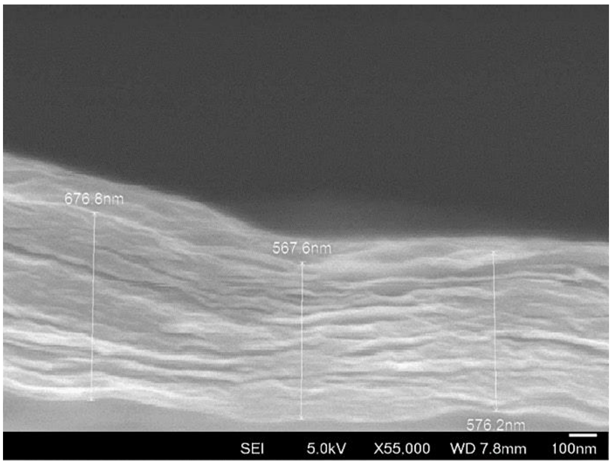

Figure S4. SEM image of a cross-section of a membrane made onto $CaSO_4/Al_2O_3$ porous plate.

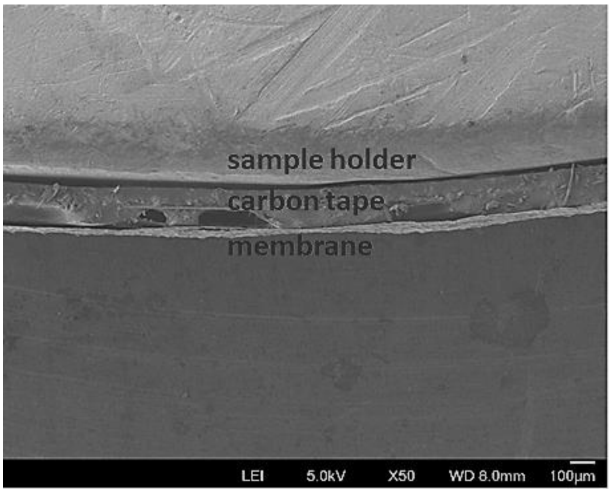

(**a**)

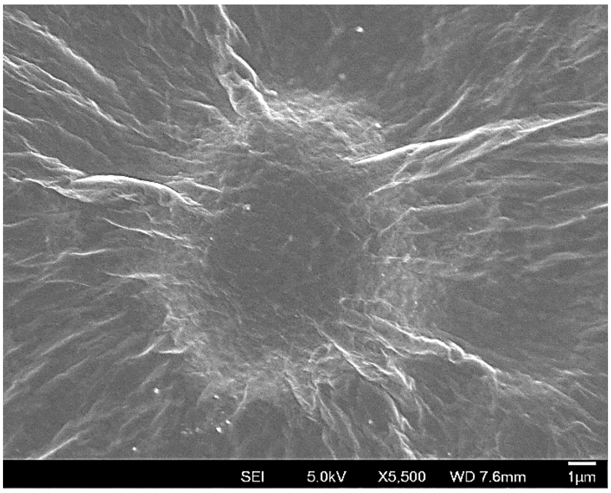

(**b**)

Figure S5. SEM image of a membrane made with GO-Nb_2O_5 (**a**) and membrane surface with circular "hillock" structure and surrounding ripples (**b**).

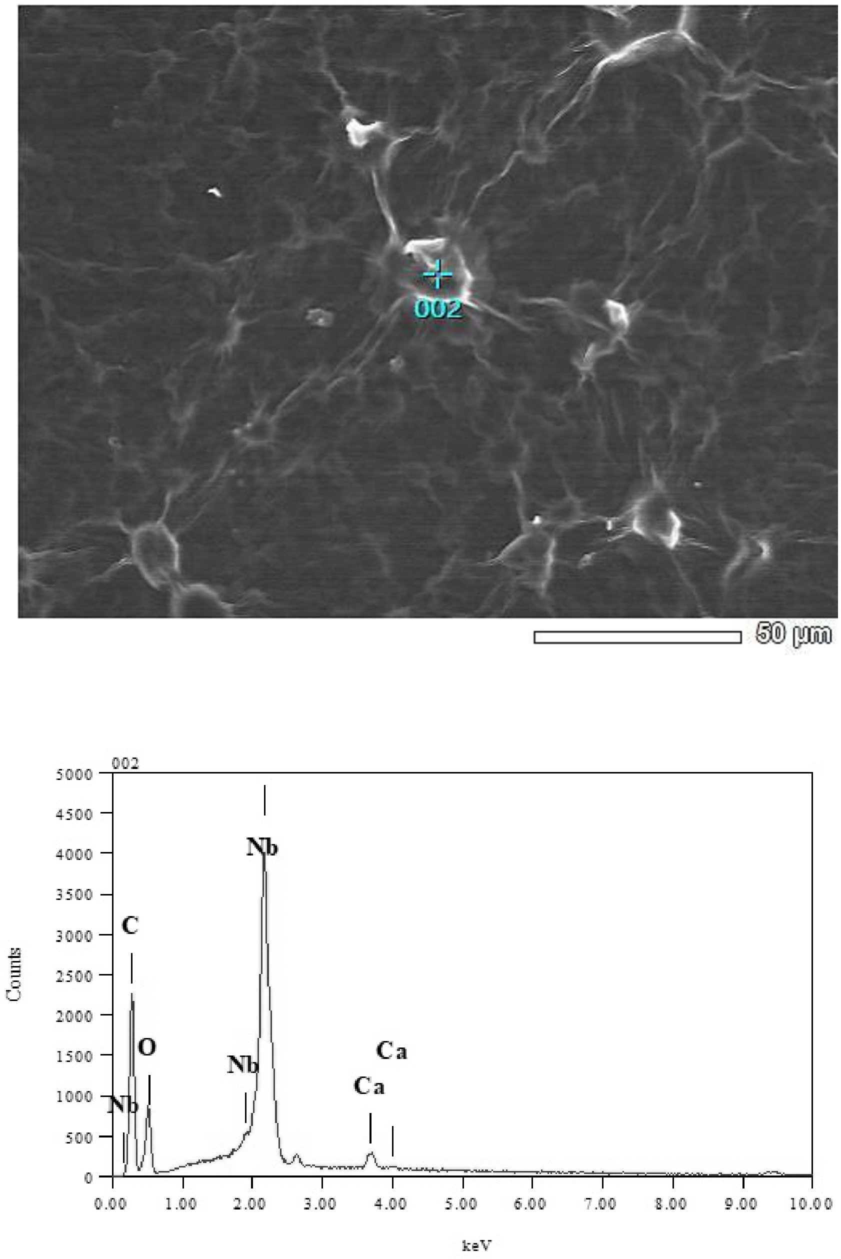

Figure S6. EDS analysis of membrane sample with Nb_2O_5 showing the main components of near-circular objects regularly distributed all over the membrane.

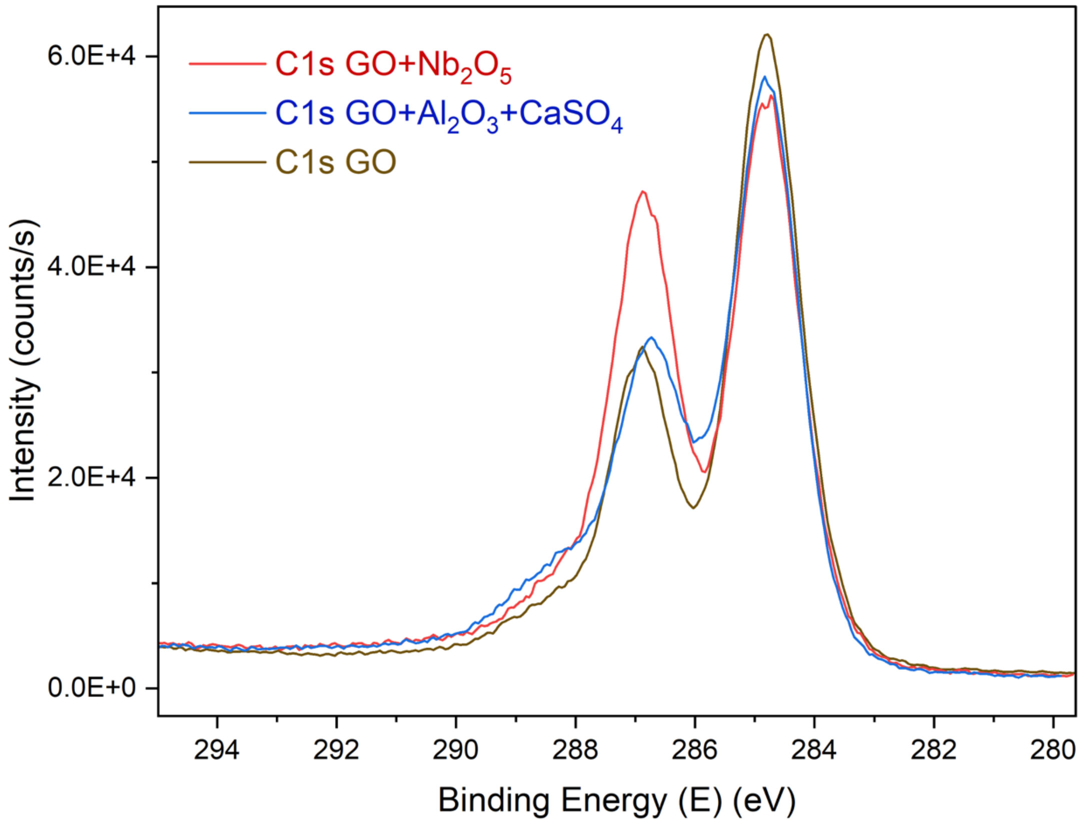

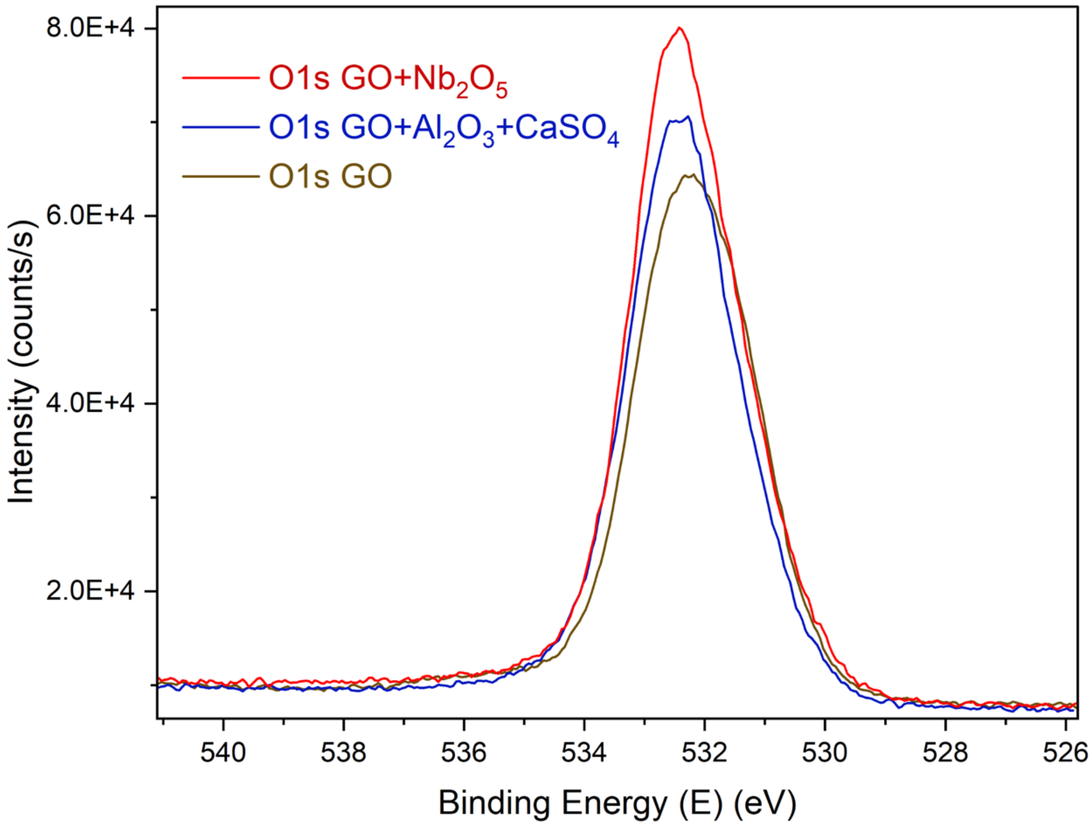

Figure S7. Qualitative comparison of the C1s and O1s spectra for samples with Nb_2O_5-based and $CaSO_4$+Al_2O_3-based cross-linking, as well as pure GO.
